# AI-supported real-time news evaluation reveals effects of time constraint on misinformation discernment

**DOI:** 10.1038/s41598-026-39555-8

**Published:** 2026-02-12

**Authors:** Yury Shevchenko, Tom Buchanan, Ulf-Dietrich Reips

**Affiliations:** 1 Research Methods, Assessment, and iScience, Department of Psychology, University of Konstanz, Universitätsstraße 10, Fach 31, Konstanz, Germany; 2 School of Social Sciences, University of Westminster, London, UK

**Keywords:** Misinformation, Experience sampling method, Ecological momentary assessment, Real-time data streaming, Artificial intelligence, Illusory truth effect, Human behaviour, Psychology and behaviour

## Abstract

This study investigates how people perceive and evaluate true and false news in their natural environment using a novel experience sampling methodology with real-time news streaming. The sample consisted of 110 participants who evaluated news headlines on their smartphones throughout the day for two weeks, receiving notifications when new content was published. The study employed a custom-developed server that captured RSS feeds from major news outlets. The server used AI (the Open AI “gpt-4-0125-preview” model) to generate modified versions of news stories on the fly, including misinformation variants. Participants evaluated news live under experimentally manipulated conditions that included time constraints for reading the news. They also provided information about their environmental context and individual characteristics. The results showed that false news items were generally rated as less accurate than true news, but this discernment decreased under time constraint. Higher digital literacy and greater satisfaction with the political system were associated with rating false news as less accurate, whereas higher dogmatism was linked to higher perceived accuracy of false news. Familiarity was also related to higher accuracy ratings for both true and false news, meaning participants rated both types of news as more accurate when they felt familiar with it, consistent with the illusory truth effect. Integrating experimental AI-guided, real-time news generation and streaming offers a novel and much-needed approach to studying misinformation perception, providing externally valid insights into how real-world factors influence people’s ability to detect and respond to false information in their daily lives.

## Introduction

In this article, we describe use of a novel experience sampling methodology to explore how context, individual differences, and time constraints affect people’s ability to discern misinformation in every-day naturalistic settings. The spread and acceptance of misinformation pose a major challenge in today’s digital environment. Most news consumption now takes place online – in contexts that frequently prioritize commercial interests over user benefit and often employ manipulative techniques to maximize profit^1^. The rapid pace of social media does not foster careful reflection, encouraging the quick consumption of “snack news”—brief headlines, short teasers, and compelling images—which can foster strong convictions rooted in illusory impressions, such as believing one is well-informed about artificial sweeteners’ health effects (e.g., aspartame causing cancer or weight gain) after repeated brief Facebook posts, despite lacking factual knowledge^2^. Many individuals forego source-checking and seldom seek additional information to verify the credibility of messages^3^.

Moreover, much of this news consumption occurs on smartphones, typically while users are multitasking, under time pressure, or without the resources or opportunity to engage deeply with content, e.g., while in bed, commuting, or even in the bathroom^4^. Prior research, mainly from laboratory and online studies, indicates that limited resources—such as time or cognitive capacity—reduce people’s ability to detect and critically evaluate misinformation^5–7^. A key contribution of this study is to examine whether these deficits are also seen in real-world naturalistic settings, measuring behavior over an extended time period closer to how people would encounter misinformation in everyday life.

Experience sampling methodology (ESM) offers a promising approach for research misinformation discernment in real-world settings. ESM involves capturing individuals’ behavior, experiences, and perceptions in real-time and in their natural environments, which can provide valuable insights into how people perceive and react to news in everyday contexts.

In this study, we assessed people’s ability to discern misinformation using a longitudinal experience sampling design in which 110 participants received three notifications per day for two weeks on their smartphones. Each notification prompted participants to evaluate a news headline streamed in real time from a major news outlet; headlines were randomly assigned to one of three conditions for each presentation: (1) the original headline, (2) a version paraphrased by AI, or (3) a version with embedded misinformation generated by AI using the GPT-4 model. For each survey, participants were randomly assigned to either a time-constrained (limited reading time) or an unconstrained (unlimited reading time) condition for viewing the news item, and reported their current context, including location, distraction, and presence of others. A baseline survey administered at the start of the study collected a range of individual characteristics such as digital literacy, political attitudes, dogmatism, cognitive reflection, religiosity, and demographic variables.

In the following theoretical introduction, we first summarize the previous research on discernment of misinformation. Then we describe the experience-sampling method and the novel implementation that we developed to study the discernment of misinformation in real-time. After that, we present the current study.

### Previous research on discernment of misinformation

The term “misinformation” is often used to collectively represent any information that turns out to be false^8^. In the literature, the concept of misinformation coexists with other terms, such as “disinformation”, emphasizing the intentional manipulation of information^9,10^. Transmission of misinformation is a part of a communication process, so factors such as receiver, message, context, and source determine whether the misinformation is going to be believed or recognized as false^11^. In the current work, we focus on characteristics of the receiver (individual traits and states), message (whether the news contain misinformation or not), and context (environment), and do not consider source characteristics. One reason to pay less attention to the source is that misinformation does not come only from “fake news” sources. Even factual news from mainstream sources (such as official news with catchy titles) can be used to promote false or misleading narratives^12^.

With respect to a receiver, previous research has identified several key cognitive mechanisms that influence how individuals process false information. Although there are many factors that affect the processing of misinformation^8,13^, we focus on the cognitive account that emphasizes that when people engage in deliberation, it usually helps to discern misinformation^5,6^. On the other hand, when people are distracted from thinking analytically, they fall for false news^14^. This empirical observation aligns with a dual-process perspective, which distinguishes between intuitive and deliberate cognitive processing^15^. Whereas intuitive processes allow for quick responses, deliberation requires effort and can override potentially incorrect intuitive responses. Why people engage in deliberation seems to be a combination of situational factors (e.g., having enough time to deliberate and not being distracted) and individual differences (e.g., tendency for analytical thinking).

With regard to situational factors, previous research has shown that people placed under time pressure are less able to discern false information^6,7^. For example, Sultan and colleagues found that time pressure specifically reduced people’s ability to distinguish true from false news, rather than altering the general tendency to judge news as true or false^7^. Bago and colleagues, by allowing participants to revise veracity decisions initially made under time pressure, found that these later, less pressured decisions were more accurate—meaning people were less likely to believe false news once they had time to deliberate^6^.

However, the role of contextual factors—such as people’s location or activities while evaluating news—that might increase distraction and susceptibility to false news has not been systematically examined. For instance, reviews and meta-analyses on misinformation judgment do not generally address such situational factors in detail^8,13^. One reason could be that the methodologies of prior studies typically did not allow for such evaluations, as most were conducted in laboratory or online settings where participants were expected to focus solely on the experimental task, not engage in other concurrent activities.

With respect to individual differences, the number of factors, such as analytical thinking, digital literacy, personality, and political orientation, was shown to contribute to the discernment of misinformation^16^. Previous research has shown that people higher in analytical thinking tend to engage in effortful, deliberate thinking and override intuitive responses with considered judgments, which should help to differentiate true and false headlines^5^. Analytical thinking, commonly measured with the cognitive reflection test (CRT), is found to be one of the strongest predictors of misinformation discernment^17^. Digital literacy – defined as knowledge about the digital environment – was found to be positively related to the ability to discern misinformation^18^. Certain aspects of personality may be related to the ability to discern misinformation. Bronstein et al.^19^ showed that scores on measures of delusion-like beliefs, dogmatism, and religious fundamentalism were associated with higher belief in false news stories. This is consistent with research that has used other measures of distorted thinking: for example, positive schizotypy has been found to be associated with poorer veracity discernment for false information^20^.

Another line of research highlights motivational reasons for believing misinformation, showing that political identification with a party can bias information processing^13,21^. This ideological belief bias means that individuals are more likely to accept information that aligns with their partisan views, and reject information that conflicts with them. Partisan congruence increases the likelihood that news is perceived as true if it matches one’s ideological beliefs. While the previous research has sometimes positioned motivational and cognitive accounts as competing explanations^6,22^, evidence indicates that both can operate simultaneously via different mechanisms^23^.

Although our primary focus is on the discernment of misinformation, it is important to recognize that misinformation research also addresses other relevant aspects, such as the sharing of misinformation, the effect of familiarity, and the impact of social engagement metrics. Findings show a discrepancy between accuracy ratings and sharing decisions—people may accurately judge news headlines as false but still choose to share them, suggesting that accuracy discernment and sharing behavior are partially independent processes^24,25^. Additionally, studies on familiarity demonstrate that repeated exposure to information, even if false, can increase the perception of its truthfulness—a phenomenon known as the illusory truth effect^26,27^. Finally, even if misinformation is not believed or shared, mere engagement—such as viewing or clicking—can influence individuals and others. Empirical evidence shows that exposure to high social engagement metrics amplifies vulnerability to low-credibility content, making people less likely to scrutinize questionable information and more likely to endorse or interact with it^28^.

### Novel method to study evaluation of misinformation in real-life

The discernment of misinformation in real-life has been relatively under-researched. There have been a few examples of platforms built to imitate the spread of misinformation through social networks^29^ or using the Social Lab^30^. However, these environments can still be perceived as artificial by participants, and the content of the messages (e.g., false news) has to be prepared by researchers in advance. Other researchers have tried to inject misinformation into Twitter posts using a custom browser extension^31^. This allowed the authors to examine interventions (such as misinformation flags and social cues) within the natural environment of Twitter, and potentially increase the external validity of findings. However, the range of manipulations that can be evaluated with this methodology is limited. Here, we present a novel variation of the experience sampling method that allows more diverse experimental manipulations, while still having greater external validity than traditional laboratory methods.

*Experience sampling methodology* (ESM): Experience sampling (also called “ambulatory assessment”, “ecological momentary assessment” or “diary studies”), examines participants’ behavior and thoughts in their natural environment. Participants nowadays often use their smartphones, where they can receive signals (e.g., push notifications) and provide responses. ESM signals can be sent at randomly chosen times or time intervals^32^ but may also be triggered by occurrence of an event of interest. With the event-contingent design, participants are asked to respond each time a specific event occurs to them (e.g., after they read a news story). However, self-initiating the report is associated with a number of problems, such as social desirability or simply forgetting to report an event^33^. With the Samply software that was developed in our research group^34^, we circumvent this problem by sending participants notifications at the moment when a news story is published. In addition, by integrating AI tools, we can modify the content of news headlines (pulled from RSS feeds of major news outlets) on the fly and present participants with different versions of the news. This streaming method can provide more insight into how people perceive misinformation in everyday contexts.

### Current study

Previous laboratory and online studies have examined how time constraints affect people’s ability to discern true from false news, typically using pre-selected news in single-session experiments^6,7^. However, these studies have not addressed whether this effect holds under more externally valid conditions—namely, when people encounter news on their smartphones throughout daily life. To address this gap, our study applied an experience sampling approach: participants evaluated news on their smartphones across the day, with news items delivered in real time instead of being pre-selected.

Unlike previous research that imposed time pressure by limiting the period for accuracy ratings^6^, we introduced a constraint on reading time (7 s per news item) but allowed unlimited time for providing an accuracy judgment afterward. This design both minimizes non-response due to failure to respond on time and more closely models real-world conditions, where people may have limited time to read the news but can reflect afterward. We argue this constraint is more externally valid, as smartphone users often skim news rapidly but can deliberate before forming opinions.

Our primary outcome was discernment of misinformation, operationalized as participants’ accuracy ratings of the news. To enrich understanding of how people engage with news, we assessed several secondary outcomes: intention to share the news, intention to read the full news story, familiarity with news items, and willingness to further engage by clicking through to the original story. These measures were selected based on prior research linking them to real-world propagation and impact of misinformation. As a quality control step, participants also reported whether they were unable to read the news. This was necessary due to potential technical issues introduced by AI-based modifications of the news items, which could affect comprehension regardless of veracity. We anticipated that participants would report lower familiarity with news items containing injected misinformation. This expectation follows from our experimental design: AI-generated misinformation was crafted to appear less recognizable than original or paraphrased versions, reducing the likelihood of prior exposure.

The current study also evaluated participants’ context and state as potential influences on their ability to discern misinformation. Specifically, we assessed self-reported situational factors such as the presence of others, noise levels, and distractions—elements that could restrict focused deliberation when processing news content. For example, encountering news in noisy or crowded environments might hinder careful scrutiny and thus impair the detection of misinformation. Additionally, we measured individual difference variables previously linked to misinformation discernment, such as analytical thinking and digital literacy, incorporating them as robust covariates in our analyses to ensure comprehensive control.

This study was designed to clarify whether a reading time constraint impairs people’s ability to detect misinformation when encountering news in real-world settings on their smartphones, and to explore whether individual or situational factors moderate this effect. Our main hypothesis was that restricting reading time would reduce accuracy in discerning false news, even when unlimited time is allowed for judgment, capturing real-world vulnerability to misinformation under everyday constraints.

## Results

### Accuracy ratings

In our experimental setup, each participant was presented with one news headline in each trial (with three trials per day, and two weeks of the study). In each trial, it was randomly decided for each participant which variant of the news they would see (the first factor: original, paraphrased, or with misinformation) and whether a time constraint was present (the second factor: with and without a time constraint). To evaluate our main hypothesis, we ran a linear mixed model with the type of news (false news vs. true news as reference, where “true news” combines both original and paraphrased versions due to no observed differences in accuracy ratings, familiarity, or readability) and time constraint (with vs. without as reference) as fixed effects. Random effects for participants and news headlines were included (see Table 1). The dependent variable was perceived accuracy, rated from 1 (“Extremely unlikely”) to 5 (“Extremely likely”), with high scores indicating greater perceived truthfulness of the headline.

**Fig. 1:**
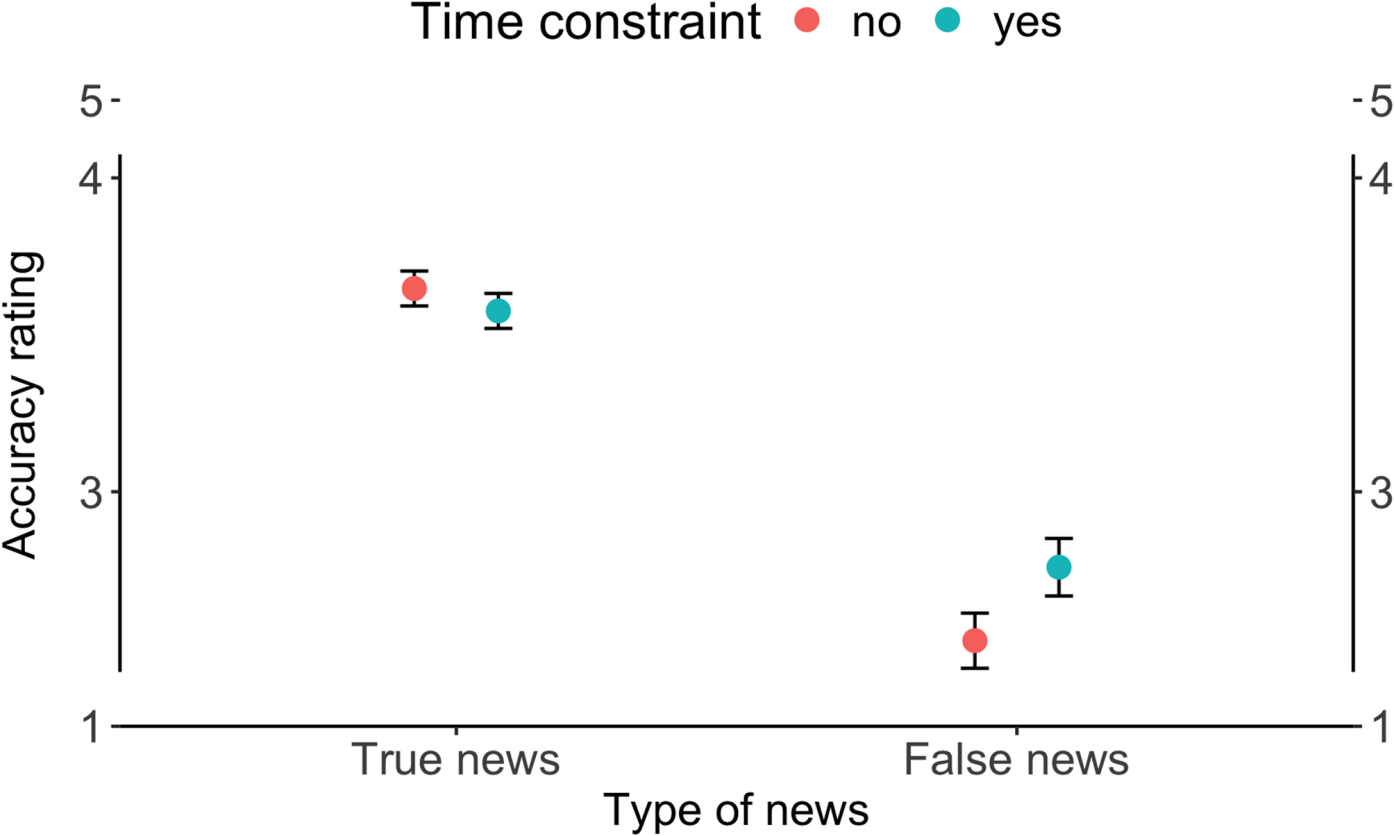
The effect of type of news and time constraint on the accuracy ratings. *Note*. Means and 95% confidence intervals are shown. The y-axes are scaled and truncated for succinctness in the visual representation.

False news was judged as significantly less accurate (*M* = 2.63, *SD* = 1.21) than true news (*M* = 3.61, *SD* = 1.07), *b* = -1.09, *SE* = 0.05, *p* < .001 (see Fig. 1). While there was no general effect of time constraint on the accuracy ratings (*b* = -0.06, *SE* = 0.04, *p* = .14), we observed a significant interaction between news type and time constraint (*b* = 0.27, *SE* = 0.07, *p* < .001). Simple-effects tests showed that time constraint did not significantly change perceived accuracy for true news (no – yes: estimate = 0.06, *SE* = 0.04, *p* = .14), whereas false news was rated as significantly more accurate under time constraint (no – yes: estimate = − 0.22, *SE* = 0.06, *p* < .001).

**Table 1:** Estimates for the variables influencing the accuracy rating.

Predictors	Estimates	SE	*p*
(Intercept)	3.65	0.04	< 0.001
False news (vs. true)	-1.09	0.05	< 0.001
With time constraint (vs. without)	-0.06	0.04	0.14
False news x With time constraint interaction	0.27	0.07	< 0.001

Note. The intercept represents the accuracy rating in the baseline condition, which is judging true news without time constraints.

**Fig. 2:**
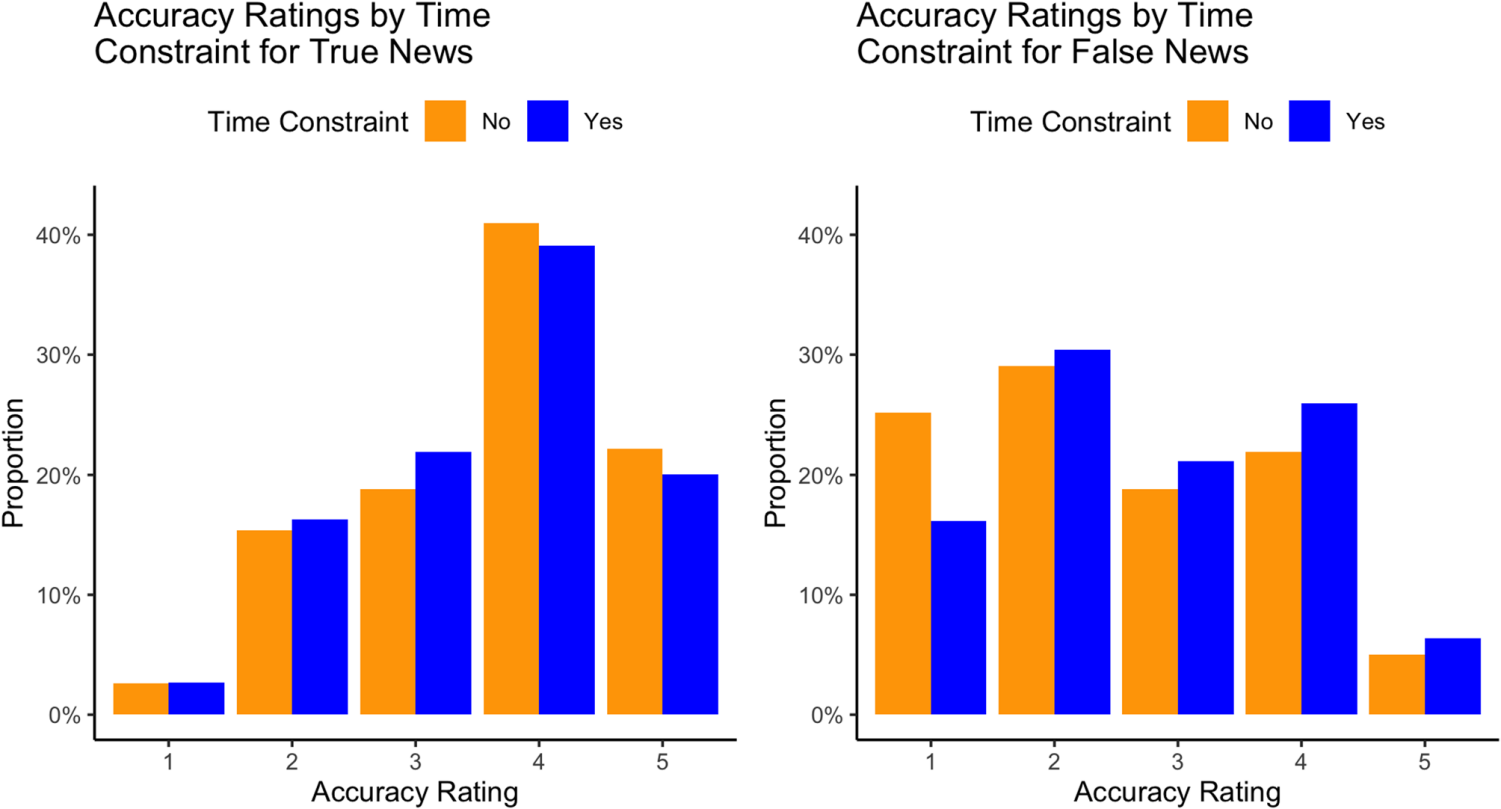
Accuracy ratings by time constraint for true and false news. *Note*. The responses are coded as: 1 – “Extremely unlikely”, 2 – “Somewhat unlikely”, 3 – “Neither likely nor unlikely”, 4 – “Somewhat likely”, 5 – “Extremely likely”.

Given the significant interaction effect between news type and time constraint, we further investigated how the responses to true and false news changed under time constraints. As can be seen from Fig. 2, for true news, under the time constraint, the probability of giving responses “Somewhat likely” and “Extremely likely” decreased. On the other hand, introduction of the time constraint in the false news condition reduced the probability of answering “Extremely unlikely” and increased the probability of giving other responses.

### Individual and situational factors

We repeated the main analysis with covariates and their interactions with news type (true vs. false) to assess individual and situational effects on misinformation discernment (see Table A1 in Appendix). Time constraint increased accuracy ratings only for false news (*b* = 0.23, *SE* = 0.07, *p* < .001), replicating the main analysis. Hearing about the news before (*b* = 1.08, *SE* = 0.07, *p* < .001) or being unsure about this (*b* = 0.51, *SE* = 0.06, *p* < .001) was related to higher accuracy ratings for both true and false news, with being unsure about prior exposure also linked to higher ratings specifically for false news (*b* = 0.36, *SE* = 0.10, *p* < .001). Higher digital literacy (*b* = -0.29, *SE* = 0.08, *p* < .001) and greater satisfaction with the political system (*b* = -0.30, *SE* = 0.08, *p* < .001) were associated with lower accuracy ratings for false news, while higher dogmatism was related to higher ratings for false news (*b* = 0.12, *SE* = 0.05, *p* = .016).

### Sharing intention, reading intention, and engagement with the news

After judging the accuracy of the news, participants indicated whether they would share it (sharing intention) and whether they would like to read more about it (reading intention). We also recorded whether they clicked on the original news link after learning whether it was true or false (engagement with the news). To assess the effects of news type and time constraint, we ran logistic mixed models with both main effects and their interaction, including random effects for participants and news headlines (see Table 2). The dependent variables were binary, where “0” indicated a negative and “1” a positive response. For sharing intention, participants were less likely to share false news (*M* = 0.07, *SD* = 0.25) than true news (*M* = 0.11, *SD* = 0.31), odds ratio = 0.46 (*SE* = 0.10, *p* < .001). There was no significant main effect of time constraint or interaction with news type. For reading intention, no significant main effects or interactions emerged (all *p*s > 0.05). Regarding engagement, participants clicked the link less often for false news (*M* = 0.07, *SD* = 0.26) than for true news (*M* = 0.09, *SD* = 0.29), odds ratio = 0.52 (*SE* = 0.11, *p* = .002). While time constraint had no significant main effect (*b* = 0.80, *SE* = 0.13, *p* = .17), there was a significant interaction with news type (*b* = 1.96, *SE* = 0.58, *p* = .024), indicating that under time constraint, false news links were clicked more often than in the no–time constraint condition.

**Table 2:** Estimates for the variables influencing the sharing intention, reading intention, and engagement with the news.

Predictors	Sharing intention			Reading intention			Engagement with the news		
	Odds Ratios	SE	*p*	Odds Ratios	SE	*p*	Odds Ratios	SE	*p*
(Intercept)	0.02	0.01	< 0.001	0.20	0.06	< 0.001	0.06	0.01	< 0.001
False news (vs. true)	0.46	0.10	< 0.001	1.09	0.16	0.54	0.52	0.11	0.002
With time constraint (vs. without)	0.72	0.12	0.05	1.04	0.12	0.71	0.80	0.13	0.17
False news x With time constraint interaction	0.98	0.33	0.95	1.05	0.21	0.82	1.96	0.58	0.024

Note. The intercept represents the ratings for true news without time constraint (the reference condition).

### Readability and familiarity

A total of 432 news items were captured during the study period, and we compared readability and familiarity across the three versions (original, paraphrased, or with embedded misinformation), with different participants viewing different versions of the same news item. Readability served as a quality check, and familiarity as a face-validity check, with the expectation that false news would be less familiar.

Ratings were aggregated by news item and condition (mean % non-readable and unfamiliar), then analyzed with ANOVAs and post-hoc tests (see Table 3 for descriptive statistics). Readability did not differ significantly between conditions, F(2, 327) = 0.18, *p* = .84. Familiarity differed significantly, F(2, 327) = 9.23, *p* < .001: misinformation headlines were less familiar than original (t = 3.67, d = 0.49, *p* < .001) and paraphrased versions (t = 3.77, d = 0.51, *p* < .001).

**Table 3:** News stories in different experimental conditions.

Condition	*N*_trials_	Number of non-readable news trials (%)	Number of trials with unfamiliar news (%)
Original news	1401	14 (0.9%)	1033 (74%)
Paraphrased news	1434	19 (1.3%)	1065 (74%)
News with misinformation	1403	16 (1.1%)	1178 (84%)
All	4238	49 (1.1%)	3276 (77%)

## Discussion

### Accuracy ratings

The primary finding of our study is that time constraint increased perceived accuracy of false news, compared to the no time constraint condition. This effect was specific: while time constraint did not alter accuracy ratings for true news, participants rated false news as significantly more accurate when they had only seven seconds to read them. This pattern is consistent with prior evidence showing that limited cognitive resources, such as when under time pressure, negatively affect the ability to discern misinformation, resulting in increased belief in false information^6,7^.

Our study extends previous research by demonstrating these effects in a naturalistic, real-world context through the use of experience sampling methodology (ESM) conducted via smartphones. Unlike earlier studies, which commonly relied on pre-selected headlines in controlled laboratory or online settings, our participants evaluated a stream of real news headlines in real time throughout the day. This approach increased external validity and allowed for the assessment of how varying situational contexts might influence responses.

A detailed analysis revealed that the impact of time constraint was evident across multiple response categories—not just a shift toward neutral or “Neither unlikely nor likely” responses, but also an increase in ratings such as “Somewhat likely” and “Extremely likely” that a false headline was accurate. One explanation for this could be that in the time constraint condition participants deliberated less and therefore could be less engaged in analytical thinking^35^. In this way, our study contributes to the literature showing that the lack of careful reasoning is associated with poorer discernment of false news^36^.

The implication of our finding is that limited time resources in everyday news consumption may indeed reduce people’s ability to distinguish between true and false information. This is especially concerning because digital environments are often designed to maximize user engagement and commercial gain^1^, which can discourage deliberation and careful reasoning on the part of users. As a result, when individuals consume news on smartphones—typically by quickly scrolling through headlines—this environment can foster misperceptions of truth. Such rapid, repeated exposure to headlines can create what some researchers have called an “illusory feeling” of truth^2^.

### Individual and situational factors

The observed relationship between prior exposure and increased perceived correctness of the news supports the illusory truth effect^37,38^. When participants reported previous exposure or uncertainty about exposure, they rated both true and false news as more correct. By streaming the news in real time, we expected a minimum degree of familiarity with the news, as the news headlines were immediately presented to participants after they were released by a news agency. Most of the news stories were indeed new for participants, which was indicated by a low degree of familiarity with the news content (77% of all news items were rated as not having been heard of before). However, people might still feel familiar with the topic of the news, or with a similar issue in the past. Also, as we could not control for the news to be exceptionally new, people might have seen news that were a follow-up of the previous event, which might be familiar to participants. Fewer people were familiar with the false news than with the true news, which may be an indicator that our real-time AI news modification algorithm worked, to a certain extent. For similar reason that familiarity could be error-prone, participants might have felt like they had already seen the false news or a similar news story before.

Among factors that specifically decreased accuracy ratings of false news (which was supported by a significant interaction effect), digital literacy played a significant role in discerning false information. This corroborates findings of Sirlin et al.^39^ that digital literacy is associated with more discerning accuracy judgments, and that digital media literacy interventions may help to improve discernment of false news^40^.

Our findings revealed that individuals reporting higher political system satisfaction demonstrated enhanced ability to identify misinformation. This aligns with Humprecht’s^41^ cross-national research, which found that politically satisfied citizens show greater resilience to disinformation, particularly in European contexts like Germany, where our study was conducted. The relationship between political satisfaction and misinformation susceptibility may be explained by the tendency of politically dissatisfied individuals to embrace conspiracy theories and anti-establishment narratives. This pattern connects to broader research on the socio-affective dimensions of misinformation engagement, including the role of personal ideology and partisan bias^13^. Indeed, recent studies have consistently shown that individuals are more likely to accept and propagate misinformation that aligns with their pre-existing ideological beliefs^42^.

Finally, the higher level of dogmatism was associated with higher ratings of accuracy for false news. This is line with previous research showing that higher dogmatism is related to higher belief in false news^19^. Personality traits such as dogmatism not only increase the likelihood of accepting false news but may also foster resistance to corrective information^43^, thereby perpetuating the spread of misinformation within social networks.

### Sharing Intention, reading Intention, and engagement with the news

Participants’ scores for sharing intention, reading interest, and further engagement with the news through clicking the news link were in general at the lower end of the scale, suggesting a general cautiousness about news engagement. This pattern is documented in the literature, such as general reluctance to share the news^44^ and the discrepancy between how people evaluate information and their willingness to share it^45^. The responses at the lower end of the scale might indicate a floor effect, where most responses clustering at the minimum value decrease the variability in the data.

Further analysis found that for the false news content the overall engagement was lower: the participants showed decreased willingness to share false news stories and were less likely to click on original news links after learning that a story was false. This behavioral pattern aligned with participants’ demonstrated ability to judge false news as less accurate than true news.

The study reveals an interesting paradox in user engagement: While participants were less likely to share false news, they showed increased interest in clicking through to original sources when presented with the false news under time constraints. The decision to click on the news link occurred after participants were informed about the news content’s false nature and were presented with both modified and original versions of the news. One explanation might be that because participants only saw the news for seven seconds, and were told that it was false, they then clicked the link in order to read it properly and see what was untrue. When there was no time pressure, and they had been able to see the news while answering the questions, they might have had no motivation to further investigate its content. This suggests that misinformation might drive engagement even when users are skeptical of its veracity, and reveals a critical challenge for content recommendation systems. The engagement metrics such as time spent and click-through rates – commonly used by algorithms to gauge content value – may inadvertently promote misinformation due to its ability to drive user interaction, even when users recognize its dubious nature.

### Methodological innovation

The study’s real-time streaming methodology represents a significant advancement over traditional laboratory or online studies. By capturing responses to news stories as they naturally occur, the research achieves higher external validity than previous studies using pre-selected news items. This approach provides insights into how people respond to misinformation in their daily lives, offering a more realistic understanding of misinformation perception and sharing behaviors.

To further enhance system effectiveness, future studies should consider transitioning from static questionnaires which have often been used in the past, to dynamic, web-based experimental platforms. Such online platforms can automate recruitment, randomize stimuli, and streamline data collection, reducing manual error and increasing sample diversity. Moreover, integrating adaptive survey designs—responsive to participant input and real-world triggers—can capture nuanced longitudinal changes in misinformation belief and sharing with greater accuracy.

The integration of experience sampling methodology (ESM) proves particularly valuable in investigating the cumulative effects of repeated misinformation exposure^27^, offering a longitudinal perspective previously difficult to achieve in controlled settings. This methodological innovation enables researchers to track the evolution of belief formation and information-sharing decisions across multiple encounters with misleading content, providing a more comprehensive understanding of how misinformation permeates through social networks in real-world contexts.

### Study limitations

In our experimental setup, the sharing decision was consistently made immediately after participants rated the accuracy of each headline. While practical, this structure may introduce a systematic bias, as participants are repeatedly prompted to consider the veracity of the content before deciding on sharing, an effect identified as an “accuracy prompt”^25^. The meta-analysis by Pennycook and colleagues demonstrates that accuracy prompts reduce sharing intentions for false news^25^. Given that our study involved repeated judgments over days, eliminating accuracy prompts entirely is not feasible—a persistent challenge for longitudinal designs. To mitigate these effects, future implementations could employ a randomized question order within each trial, so that sometimes sharing decisions precede accuracy ratings. Although such randomization might reduce systematic bias to some extent, mere participation in a misinformation evaluation study may itself serve as a de facto accuracy prompt, priming individuals to scrutinize information more closely than they might in everyday contexts. This suggests that our findings may underestimate the strength of the real effects.

This limitation relates to another key aspect of our design: revealing the true nature of the news to participants. We chose to debrief participants at the end of each trial, both for ethical and practical reasons. Ethically, immediate debriefing can minimize any potential long-term detrimental effects of exposure to misinformation^46^. Practically, tracking all presented headlines and delaying debriefing until the study conclusion was not feasible. Because each headline was randomly selected and differed every time, and because a random mechanism determined whether a particular headline contained misinformation, we reasoned that learning the true nature of one headline would not systematically influence judgments about subsequent headlines. Nevertheless, it remains possible that repeated exposure and immediate debriefing could incidentally enhance participants’ ability to discern false from true news over time^47^.

Regarding sample characteristics, our reliance on a student sample—particularly one with a high proportion of female participants—may introduce sampling bias and limit generalizability. Future research should aim to replicate and extend our findings, possibly by using the multiple site entry technique^48^. A further limitation concerns the distinct sub-samples: participants were exposed to either German or English news headlines sourced from different media channels. Although our analyses did not reveal systematic differences between the German and English sub-samples, potential influences stemming from language, media source, or cultural context may still exist and were possibly undetected in the current study. Upcoming investigations should include larger, more balanced cross-linguistic groups and consider multi-site sampling to better assess the impact of language and media source on misinformation evaluation and sharing behaviors.

### Future research

Cognitive constraints, individual differences, and contextual elements intricately shape how people process misinformation, indicating that successful countermeasures must target multiple facets of information assessment. Our research points to the advantages of longitudinal studies examining exposure to false information in naturalistic environments. We investigated the impact of lack of deliberation on the evaluation of misinformation, following an idea that under time constraints people are more susceptible to misinformation. Future research may adopt the experience sampling method to test other lab-derived theoretical predictions in the field. Moreover, future studies can still implement experimental manipulations with the content or the source of information. Altogether, understanding how people evaluate and share misinformation in real life should help to yield more refined, context-specific strategies to combat its dissemination.

## Methods

### Participants

Data were collected in the period between February 24, 2024 and May 11, 2024. The participants were students of the University of Konstanz recruited from the participant management platform Sona Systems. Participation was reimbursed with course credit. Additionally, a lottery was organized for the participants who completed over 80% of surveys, with the chance to win one of ten 50€ vouchers (58 US dollars (according to exchange rate on the 5th of August 2025)). We collected data from 110 participants, 96 women, 11 men, and 3 unknown, *M*_*age*_ *(SD)* = 22 (3.71), who participated in at least one daily survey. Based on their preferred language, 93 participants completed the German version, and 17 participants completed the English version of the study. On average, participants completed 39 surveys (*SD* = 11.22), which corresponds to 93% of all daily surveys. Overall, 432 news headlines were processed during the data collection. We removed trials where participants responded to the same stimulus more than once (*n* = 100, 2%), trials where participants left the survey before the last screen (*n* = 80, 2%), and the trials with non-readable news (*n* = 49, 1%). This left 4189 trials that were used for data analysis, from 4418 that were initially recorded. Dropout rates after presentation of news did not differ between the condition with and without time constraint, *N*_*time constraint*_ = 25 vs. *N*_*no time constraint*_ = 29, *X*^*2*^ (1) = 0.30, *p* = .59.

### Procedure

The study lasted for fifteen days for each participant. Participants who clicked on the study link were redirected to a webpage containing the baseline survey. Here, they were informed about the study’s objectives, provided with an informed consent form, and asked to complete the baseline survey, which collected information on socio-demographic variables and individual differences. They were then instructed on how to install and use the Samply Research mobile application. To anonymously connect participants’ data across different surveys, each participant was shown a unique anonymous code in the baseline survey, which they entered into the mobile application. This participant code was subsequently recorded in all surveys completed through the mobile app. After joining the study in the mobile app, participants immediately received a test notification to ensure proper functionality. The sampling schedule of daily surveys followed an event-contingent design, where the notifications were sent three times a day immediately after the news was published in the RSS feed. At the end of Day 15, marking the end of the study, participants received a link to a debriefing survey, in which they were asked for feedback and instructed how to leave the study and delete the mobile application.

### Research design

**Fig. 3:**
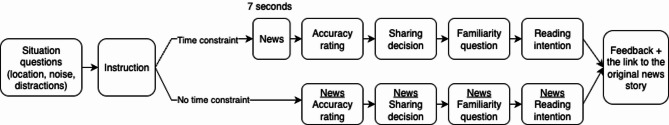
Research design: time-constraint vs. no-time-constraint conditions *Note.* In the time-constraint condition, the news is presented for only 7 s, and then participants have to answer the questions. In the no-time-constraint condition, the news is always presented next to the questions.

The presence of the time constraint and the type of the news message (original, paraphrased, and with misinformation) in the daily surveys were manipulated in a within-subject design, with random assignment for each survey (see Fig. 3). In the time constraint condition, participants were presented with the news headline for 7 s. After that, the screen changed to the questions. We did not put any time constraints on the judgment itself, in order to avoid non-response cases. In the no constraint condition, the news were always presented next to the questions and participants had unlimited time to provide their judgments. The news content manipulation presented participants with either the original news text, a paraphrased version, or a version containing misinformation, with the paraphrased condition serving as a control for possible effects of AI text processing.

### Streaming of the news to participants

We programmed a server-based NodeJS application (available at GitHub https://github.com/Yury-Shevchenko/samply-streamer) to scan and process updates from a specific RSS feed, triggering notifications using a POST request to the Samply API^49^. The core functions of the application include periodically scanning the RSS feed using the npm package “rss-feed-emitter”^50^ at a one-minute refresh rate, processing and modifying news messages in one of the experimental conditions, storing the messages in a MongoDB database, and sending a POST request through the fetch command.

For this study, we selected the RSS feed of the German news agency *Tagesschau* (tagesschau.de - Die Nachrichten der ARD) for German-speaking participants and the *BBC News* for English-speaking participants. Given that these feeds publish numerous news items daily and news can be released at any time, we defined three time windows during which only the first news message was processed: 9:00–13:00, 13:00–17:00, and 17:00–21:00 local time of the participant. This approach ensured that only three news items per day were sent to participants.

A typical news message pulled from the RSS feed consisted of a header and news content (maximum of three sentences). The text of the message was pre-processed with the npm package “text-cleaner” to remove HTML^51^. After that, we used the Chat-GPT API (OpenAI model “gpt-4-0125-preview”) to modify the message to either contain misinformation or a paraphrased version of the original text (see the prompt below). Once the original and modified versions were saved in the database, a POST request was sent to the Samply API. To avoid transferring the news text directly through the notification, the server sent a web link that was generated for each text. When participants opened a survey by tapping on the notification (which did not contain a news message), the text of the news was automatically retrieved from that web link and displayed to participants. For each survey, participants were randomly assigned to view either the original unmodified version, a paraphrased version, or a version of the news text that included misinformation.

### Prompt for Chat-GPT AI

“We are conducting a study on the perception of fake news. For this particular situation, you need to come up with two short (maximum 3 sentences) modified messages based on the original message, which will be provided. The language of the modified messages should match the language of the original message. So if the original message is in English, the modified messages should be in English. If the original message is in German, the modified messages should be in German. The modified messages should have the same length as the original message. The first modified message should be as close as possible to original news, so it should not contain misinformation. The second modified message should be fake news, so it should contain misinformation. You must respond with only the text for these two messages. Do not include any headers for modified messages. The original message is”:

### Measures

All surveys were programmed in the lab.js experiment builder^52^ and hosted on the Open Lab platform for data collection^53^.

#### Baseline survey

The baseline survey collected information on socio-demographic variables and individual differences (e.g., dogmatism, digital literacy, cognitive reflection).

##### Dogmatism

Dogmatism was measured using the 22-item DOG Scale^54^. Participants rated their agreement with each statement on a 9-point scale ranging from − 4 (strongly disagree) to 4 (strongly agree).

##### Schizotypy

To measure tendencies towards delusion-like ideation, we used the cognitive-perceptual dimension of the Schizotypal Personality Questionnaire – Brief Revised Updated^55^. This instrument evaluates schizotypy, a collection of traits that resemble the disordered thinking seen in schizophrenia. The study utilized 14 items from the cognitive perceptual subscale, which measures suspiciousness, ideas of reference, magical thinking, and unusual perceptions. These are all aspects of ‘positive schizotypy’. Participants rated their agreement with each statement on a 5-point Likert scale from “Strongly disagree” to “Strongly agree”.

##### Religious schema scale

The Religious Schema Scale was employed to measure religious attitudes^56^. The 15-items scale consists of three subscales: Truth of Texts & Teachings (ttt), Xenosophia, and Fairness, Tolerance and Rational Choice (ftr). Participants rated their agreement with each statement on a 5-point Likert scale ranging from “strongly agree” to “strongly disagree”. The ttt subscale was of primary interest for this study due to its strong positive correlation with Altemeyer and Hunsberger’s^57^ Religious Fundamentalism Scale, which has been shown to be related to the sharing of fake news^19^.

##### Cognitive reflection

Cognitive reflection was measured using two instruments: The original Cognitive Reflection Test ^[CRT58^ and its newer variant, “CRT-2”^59^. The inclusion of CRT-2 helped address potential score inflation due to participant familiarity with the original CRT^60^.

##### Digital literacy

Digital literacy was assessed using an adapted version of Hargittai’s Internet Skill Scale^61^, updated by Guess and Munger^62^. This 21-item scale presented participants with internet-related terms (e.g., “app”, “hashtag”, “wiki”) and two fictitious terms (“fitibly” and “proxypod”). Participants rated their familiarity with each term on a 5-point Likert scale from “no understanding” to “full understanding”. For the German subsample, terms like “preference setting”, “advanced search”, and “tagging” were translated to their German equivalents.

##### Political orientation and political system satisfaction

Political orientation was measured on a 10-point Likert scale from “left” to “right”, following Grünhage and Reuter’s^63^ methodology. Satisfaction with the political system was evaluated using the 4-item German Satisfaction with the Political System Short Scale^64^. Participants rated their agreement with four statements about the political system on a 4-point scale from 1 (strongly approve) to 4 (strongly disapprove).

##### Online sharing behaviors

To assess participants’ online sharing behaviors, we employed two measures. First, we asked participants to indicate how likely they are generally to share political news online, using a five-point Likert scale ranging from “Extremely unlikely” to “Extremely likely”. This single-item measure provided a direct assessment of participants’ propensity to disseminate political content. For exploratory purposes, we also included a question about the types of information participants usually share online. This question offered multiple response options, including personal life updates, photos and videos, work or professional achievements, and other categories of content.

#### Daily survey

In the daily surveys, participants answered questions about their current environment and evaluated the presented news headlines. They reported on their location (e.g., at home, at family’s/friend’s place, public space), the presence of others (e.g., alone, with family members, with friends), noise level (on a six-point scale from “Silent” to “Extremely noisy”), and distraction level (none, one, or multiple). After assessing these contextual factors, participants were presented with a news item and asked to evaluate it. They rated the perceived correctness of the headline with five options from “Extremely likely” to “Extremely unlikely”, indicated their intention to share the news online (with options “Yes”, “Maybe”, or “No”), reported their familiarity with the news (“Yes”, “Unsure”, “No”, or “The text is not possible to read”, which was used in case of a technical failure), and expressed their interest in reading the full story (“Yes” or “No”). At the conclusion of each survey, the true nature of the presented news was revealed (whether it was an original text from RSS feeds; paraphrased; or contained misinformation). In the cases where the news was modified, both original and modified versions were presented next to each other. Participants were then offered a link to the original story on the source news website, and the system recorded whether they clicked on this link.

#### Debriefing survey

In the final survey at the end of the study, participants were asked if they missed any notifications and if there were any technical or other problems during the study. Open-ended questions allowed participants to give us their feedback on the study. Finally, participants were asked to enter their email address if they wished to participate in the lottery.

### Data protection and ethical approval

In this study, we implemented comprehensive measures to ensure ethical data collection and participant privacy. All methods were carried out in accordance with relevant guidelines and regulations. The experimental protocol was approved by the Ethics Committee of the University of Konstanz, according to the University’s and national statutes in Germany (RefNo: IRB25KN001-05/w). Self-report measures, which posed no risk to participants and had been validated in previous studies, were used throughout. Participants were informed of their right to withdraw from the study at any time. Informed consent was obtained through two channels: via the Samply Research mobile application for app-collected data, and through online surveys for survey-specific data. To maintain anonymity while connecting data from different sources, we employed anonymous participant codes. Personal email addresses used for app login and those provided for the lottery were stored separately from survey data. The link between emails and survey data was destroyed post-study, and lottery-related emails were deleted after prize distribution. This approach allowed us to protect participant privacy while still enabling necessary data linkage across different parts of the study.

### Statistical analysis

We employed mixed models to account for the nested structure of our data, where news headlines were clustered within participants. Finding no significant differences between original and paraphrased conditions across all dependent variables, we combined them into a single “true news” condition. To evaluate the main hypothesis, we ran a linear mixed model with the type of news (true vs. false) and time constraint (with vs. without) as fixed effects, and random effects for participants and news headlines. The dependent variable was perceived accuracy (1=“Extremely unlikely” to 5=“Extremely likely”; higher scores indicate greater perceived truthfulness of the news headline). We repeated the main analysis with covariates and their interactions with news type (true vs. false) to assess individual and situational effects on misinformation discernment. To assess the effects of news type and time constraint on the three additional dependent variables (sharing intention, reading intention, and engagement with the news), we ran logistic mixed models with both main fixed effects and their interaction, including random effects for participants and news headlines. The dependent variables were binary, where “0” indicated a negative and “1” a positive response. Finally, we used ANOVA and planned contrasts analyses to compare news items across experimental conditions in terms of their readability and familiarity to ensure balanced stimulus materials. The data and analysis script are available at OSF (https://osf.io/c3drv/).

## Data Availability

The data and analysis script are available at OSF (https://osf.io/c3drv/).

## Appendix

See Table 4.

**Table 4:** Estimates for the individual and situational variables influencing the accuracy Rating.

Predictors	Estimates	SE	*p*
(Intercept)	3.27	0.26	**< 0.001**
False news (vs. true)	-1.17	0.28	**< 0.001**
With time constraint (vs. without)	-0.06	0.04	0.14
False news x With time constraint interaction	0.23	0.07	**0.001**
Heard this news before (not sure vs. no)	0.51	0.06	**< 0.001**
Heard this news before (yes vs. no)	1.08	0.07	**< 0.001**
Level of noise	0.05	0.02	0.053
Distraction	-0.05	0.04	0.16
Being alone vs. being with other people	0.02	0.05	0.63
Public location vs. non-public location	-0.03	0.06	0.61
Age	0.01	0.01	0.54
Male vs. Female	0.12	0.11	0.30
Dogmatism score	-0.02	0.04	0.66
Schizotypy score	-0.00	0.06	0.99
Religious schema scale - Truth of Texts & Teachings subscale	0.01	0.04	0.81
Digital literacy score	0.06	0.08	0.41
Political orientation	-0.02	0.03	0.64
Satisfaction with political system	0.01	0.08	0.93
Sharing political information online	0.06	0.03	0.07
Cognitive reflection score	0.02	0.02	0.27
German vs. English language	-0.09	0.11	0.41
False news × Heard this news before (not sure)	0.36	0.10	**< 0.001**
False news × Heard this news before (yes)	-0.22	0.19	0.25
False news × Level of noise	-0.04	0.04	0.29
False news × Distraction	0.10	0.06	0.099
False news × Being alone	-0.04	0.08	0.66
False news × Public location	-0.10	0.10	0.33
False news × Age	0.02	0.01	0.065
False news × Male	-0.22	0.11	0.05
False news × Dogmatism score	0.12	0.05	**0.016**
False news × Schizotypy score	0.03	0.06	0.57
False news × Religious schema scale - Truth of Texts & Teachings subscale	-0.05	0.05	0.26
False news × Digital literacy score	-0.29	0.08	**< 0.001**
False news × Political orientation	0.05	0.03	0.17
False news × Satisfaction with political system	-0.30	0.08	**< 0.001**
False news × Sharing political information online	-0.04	0.03	0.22
False news × Cognitive reflection score	-0.03	0.02	0.17
False news × German language	-0.16	0.11	0.14

Note. The intercept represents the ratings for true news without time constraint (the reference condition). Continuous participant-level covariates (digital literacy, political system satisfaction, dogmatism, schizotypy, CRT, political orientation, political information sharing, and RSS truthfulness scores) were grand-mean centered (centered on the participant-level mean across participants) prior to analysis; thus, main effects are interpreted at the sample mean of these covariates.

## References

[1] Kozyreva, A., Lewandowsky, S. & Hertwig, R. Citizens versus the internet: confronting digital challenges with cognitive tools. *Psychol. Sci. Public. Interest.* **21**, 103–156 (2020). 33325331 10.1177/1529100620946707 PMC7745618

[2] Schäfer, S. Illusion of knowledge through Facebook news? Effects of snack news in a news feed on perceived knowledge, attitude strength, and willingness for discussions. *Comput. Hum. Behav.* **103**, 1–12 (2020).

[3] Bakshy, E., Messing, S. & Adamic, L. A. Exposure to ideologically diverse news and opinion on Facebook. *Science* **348**, 1130–1132 (2015). 25953820 10.1126/science.aaa1160

[4] [Chart of the week] Smartphones. Where do you read your news? *FIPP* (2017). https://www.fipp.com/news/chart-of-the-week-where-people-view-news/

[5] Pennycook, G. & Rand, D. G. Lazy, not biased: susceptibility to partisan fake news is better explained by lack of reasoning than by motivated reasoning. *Cognition* **188**, 39–50 (2019). 29935897 10.1016/j.cognition.2018.06.011

[6] Bago, B., Rand, D. G. & Pennycook, G. Fake news, fast and slow: deliberation reduces belief in false (but not true) news headlines. *J. Exp. Psychol. Gen.* **149**, 1608–1613 (2020). 31916834 10.1037/xge0000729

[7] Sultan, M. et al. Time pressure reduces misinformation discrimination ability but does not alter response bias. *Sci. Rep.* **12**, 22416 (2022). 36575232 10.1038/s41598-022-26209-8 PMC9794823

[8] Chen, S., Xiao, L. & Kumar, A. Spread of misinformation on social media: what contributes to it and how to combat it. *Comput. Hum. Behav.* **107643**10.1016/j.chb.2022.107643 (2022).

[9] Adams, Z., Osman, M., Bechlivanidis, C. & Meder, B. Why) is misinformation a problem? *Perspect. Psychol. Sci.* **18**, 1436–1463 (2023). 36795592 10.1177/17456916221141344 PMC10623619

[10] Kapantai, E., Christopoulou, A., Berberidis, C. & Peristeras, V. A systematic literature review on disinformation: toward a unified taxonomical framework. *New. Media Soc.* **23**, 1301–1326 (2021).

[11] Cobley, P. & Schulz, P. J. *Theories and Models of Communication* (Walter de Gruyter, 2013).

[12] Goel, P., Green, J., Lazer, D. & Resnik, P. Mainstream news articles co-shared with fake news buttress misinformation narratives. *ArXiv*10.48550/ArXiv.2308.06459 (2023).

[13] Ecker, U. K. H. et al. The psychological drivers of misinformation belief and its resistance to correction. *Nat. Rev. Psychol.* **1**, 13–29 (2022).

[14] Kwek, A., Peh, L., Tan, J. & Lee, J. X. Distractions, analytical thinking and falling for fake news: A survey of psychological factors. *Humanit. Soc. Sci. Commun.* **10**, 319 (2023). 37333884 10.1057/s41599-023-01813-9 PMC10259813

[15] Evans, J., St., B. T. & Stanovich, K. E. Dual-process theories of higher cognition: advancing the debate. *Perspect. Psychol. Sci.* **8**, 223–241 (2013). 26172965 10.1177/1745691612460685

[16] Buchanan, T. Why do people spread false information online? The effects of message and viewer characteristics on self-reported likelihood of sharing social media disinformation. *PLOS ONE*. **15**, e0239666 (2020). 33027262 10.1371/journal.pone.0239666 PMC7541057

[17] Sultan, M. et al. Susceptibility to online misinformation: A systematic meta-analysis of demographic and psychological factors. *Proc. Natl. Acad. Sci. USA* **121**, e2409329121 (2024). 10.1073/pnas.2409329121 PMC11588074 39531500

[18] Borges-Tiago, T., Tiago, F., Silva, O., Guaita Martínez, J. M. & Botella-Carrubi, D. Online users’ attitudes toward fake news: implications for brand management. *Psychol. Mark.* **37**, 1171–1184 (2020).

[19] Bronstein, M. V., Pennycook, G., Bear, A., Rand, D. G. & Cannon, T. D. Belief in fake news is associated with delusionality, dogmatism, religious fundamentalism, and reduced analytic thinking. *J. Appl. Res. Mem. Cogn.* **8**, 108–117 (2019).

[20] Buchanan, T., Maras, K. & Dando, C. Individual differences in detecting and sharing misinformation: positive schizotypy, conspiracy beliefs, and autism. *Personal Individ Differ.* **233**, 112946 (2025).

[21] Van Bavel, J. J. & Pereira, A. The partisan brain: an identity-based model of political belief. *Trends Cogn. Sci.* **22**, 213–224 (2018). 29475636 10.1016/j.tics.2018.01.004

[22] Ludwig, J. & Sommer, J. Mindsets and politically motivated reasoning about fake news. *Motiv Emot.* **48**, 249–263 (2024).

[23] Batailler, C., Brannon, S. M., Teas, P. E. & Gawronski, B. A signal detection approach to Understanding the identification of fake news. *Perspect. Psychol. Sci.* **17**, 78–98 (2022). 34264150 10.1177/1745691620986135

[24] Ceylan, G., Anderson, I. A. & Wood, W. Sharing of misinformation is habitual, not just lazy or biased. *Proc. Natl. Acad. Sci.* **120**, e2216614120 (2023). 36649414 10.1073/pnas.2216614120 PMC9942822

[25] Pennycook, G. & Rand, D. G. Accuracy prompts are a replicable and generalizable approach for reducing the spread of misinformation. *Nat. Commun.* **13**, 2333 (2022). 35484277 10.1038/s41467-022-30073-5 PMC9051116

[26] Dechêne, A., Stahl, C., Hansen, J. & Wänke, M. The truth about the truth: A meta-analytic review of the truth effect. *Personal Soc. Psychol. Rev.* **14**, 238–257 (2010). 10.1177/1088868309352251 20023210

[27] Udry, J. & Barber, S. J. The illusory truth effect: A review of how repetition increases belief in misinformation. *Curr. Opin. Psychol.* **56**, 101736 (2024). 38113667 10.1016/j.copsyc.2023.101736

[28] Avram, M., Micallef, N., Patil, S. & Menczer, F. Exposure to social engagement metrics increases vulnerability to misinformation. *Harv. Kennedy Sch. Misinformation Rev.*10.37016/mr-2020-033 (2020).

[29] Butler, L. H. et al. The (mis)information game: A social media simulator. *Behav. Res. Methods*. **56**, 2376–2397 (2024). 37433974 10.3758/s13428-023-02153-x PMC10991066

[30] Garaizar, P. & Reips, U. D. Build your own social network laboratory with social lab: A tool for research in social media. *Behav. Res. Methods*. **46**, 430–438 (2014). 24061930 10.3758/s13428-013-0385-3

[31] Jones, C. M. et al. Impact of social reference cues on misinformation sharing on social media: series of experimental studies. *J. Med. Internet Res.* **25**, e45583 (2023). 37616030 10.2196/45583 PMC10485706

[32] Shevchenko, Y. & Reips, U. D. How to prepare and conduct an experience sampling study via mobile phones. *SAGE Res. Methods Doing Res. Online*. 10.4135/9781529610116 (2022).

[33] Hultgren, B. A., Scaglione, N. M., Buben, A. & Turrisi, R. Examining protocol compliance and self-report congruence between daily diaries and event-contingent ecological momentary assessments of college student drinking. *Addict. Behav.* **110**, 106471 (2020). 32526551 10.1016/j.addbeh.2020.106471 PMC7919385

[34] Shevchenko, Y., Kuhlmann, T., Reips, U. D. & Samply A user-friendly smartphone app and web-based means of scheduling and sending mobile notifications for experience-sampling research. *Behav. Res. Methods*. **53**, 1710–1730 (2021). 33528818 10.3758/s13428-020-01527-9 PMC8367917

[35] Nurse, M. S., Ross, R. M., Isler, O. & Van Rooy, D. Analytic thinking predicts accuracy ratings and willingness to share COVID-19 misinformation in Australia. *Mem. Cognit*. **50**, 425–434 (2022). 34453286 10.3758/s13421-021-01219-5 PMC8395380

[36] Pennycook, G. & Rand, D. G. The psychology of fake news. *Trends Cogn. Sci.* **25**, 388–402 (2021). 33736957 10.1016/j.tics.2021.02.007

[37] Hassan, A. & Barber, S. J. The effects of repetition frequency on the illusory truth effect. *Cogn. Res. Princ Implic*. **6**, 38 (2021). 33983553 10.1186/s41235-021-00301-5 PMC8116821

[38] Pan, W. & Hu, T. Y. More familiar, more credible? Distinguishing two types of familiarity on the truth effect using the drift-diffusion model. *J. Soc. Psychol.* 1–19. 10.1080/00224545.2024.2363366 (2024). 10.1080/00224545.2024.2363366 38852171

[39] Sirlin, N., Epstein, Z., Arechar, A. A. & Rand, D. G. Digital literacy is associated with more discerning accuracy judgments but not sharing intentions. *Harv. Kennedy Sch. Misinf. Rev.*10.37016/mr-2020-83 (2021).

[40] Guess, A. et al. A digital media literacy intervention increases discernment between mainstream and false news in the united States and India. *Proc. Natl. Acad. Sci. U S A*. **117**, 15536–15545 (2020). 32571950 10.1073/pnas.1920498117 PMC7355018

[41] Humprecht, E. The role of trust and attitudes toward democracy in the dissemination of disinformation—a comparative analysis of six democracies. *Digit. J.* 1–18. 10.1080/21670811.2023.2200196 (2023).

[42] Schwalbe, M. C., Joseff, K., Woolley, S. & Cohen, G. L. When politics Trumps truth: political concordance versus veracity as a determinant of believing, sharing, and recalling the news. *J. Exp. Psychol. Gen.* **153**, 2524–2551 (2024). 39388117 10.1037/xge0001650

[43] Schulz, L., Rollwage, M., Dolan, R. J. & Fleming, S. M. Dogmatism manifests in Lowered information search under uncertainty. *Proc. Natl. Acad. Sci. U S A*. **117**, 31527–31534 (2020). 33214149 10.1073/pnas.2009641117 PMC7733856

[44] Yang, F. & Horning, M. Reluctant to share: how third person perceptions of fake news discourage news readers from sharing real news on social media. *Soc. Media Soc.* **6**, 2056305120955173 (2020).

[45] Pennycook, G. et al. Shifting attention to accuracy can reduce misinformation online. *Nature* **592**, 590–595 (2021). 33731933 10.1038/s41586-021-03344-2

[46] Greene, C. M. et al. Best practices for ethical conduct of misinformation research. *Eur Psychol* (2022).

[47] Greene, C. M. & Murphy, G. Debriefing works: successful Retraction of misinformation following a fake news study. *PloS One*. **18**, e0280295 (2023). 36662686 10.1371/journal.pone.0280295 PMC9858761

[48] Reips, U.-D. Web-based research in psychology. *Z. Psychol.* **229**(4), 198–213. 10.1027/2151-2604/a000475 (2021).

[49] Shevchenko, Y., Reips, U. D. & Samply Stream, A. P. I. The AI-enhanced method for real-time event data streaming. (2024). 10.3758/s13428-025-02634-1 PMC11914333 40097774

[50] Deschamps, F. rss-feed-emitter (Version 3.2.3). (2023).

[51] Gamble-Milner A. text-cleaner (Version 1.2.1). (2024).

[52] Henninger, F., Shevchenko, Y., Mertens, U. K., Kieslich, P. J. & Hilbig, B. E. lab.js: A free, open, online study builder. *Behav. Res. Methods*. **54**, 556–573 (2022). 34322854 10.3758/s13428-019-01283-5 PMC9046347

[53] Shevchenko, Y. & Open Lab A web application for running and sharing online experiments. *Behav. Res. Methods*. **1–8**10.3758/s13428-021-01776-2 (2022). 10.3758/s13428-021-01776-2 PMC9729124 35233751

[54] Altemeyer, B. Dogmatic behavior among students: testing a new measure of dogmatism. *J. Soc. Psychol.* **142**, 713–721 (2002). 12450346 10.1080/00224540209603931

[55] Davidson, C. A., Hoffman, L. & Spaulding, W. D. Schizotypal personality questionnaire – brief revised (updated): an update of norms, factor structure, and item content in a large non-clinical young adult sample. *Psychiatry Res.* **238**, 345–355 (2016). 27086255 10.1016/j.psychres.2016.01.053 PMC4834869

[56] Streib, H., Hood, R. W. & Klein, C. The religious schema scale: construction and initial validation of a quantitative measure for religious styles. *Int. J. Psychol. Relig.* **20**, 151–172 (2010).

[57] Altemeyer, B. & Hunsberger, B. A revised religious fundamentalism scale: the short and sweet of it. *Int. J. Psychol. Relig.* **14**, 47–54 (2004).

[58] Frederick, S. Cognitive reflection and decision making. *J. Econ. Perspect.* **19**, 25–42 (2005).

[59] Thomson, K. S. & Oppenheimer, D. M. Investigating an alternate form of the cognitive reflection test. *Judgm. Decis. Mak.* **11**, 99–113 (2016).

[60] Stieger, S. & Reips, U. D. A limitation of the cognitive reflection test: familiarity. *PeerJ* **4**, e2395 (2016). 27651989 10.7717/peerj.2395 PMC5018679

[61] Hargittai, E. Survey measures of web-oriented digital literacy. *Soc. Sci. Comput. Rev.* **23**, 371–379 (2005).

[62] Guess, A. M. & Munger, K. Digital literacy and online political behavior. *Polit Sci. Res. Methods*. **11**, 110–128 (2023).

[63] Grünhage, T. & Reuter, M. Political orientation is associated with behavior in public-goods- and trust-games. *Polit Behav.* **44**, 23–48 (2022).

[64] Dentler, K., Bluemke, M. & Gabriel, O. W. German satisfaction with the political system short scale (SPS). *ZIS— Collect. Items Scales Soc. Sci.*10.6102/zis278 (2020).

