# Correction: AI-supported real-time news evaluation reveals effects of time constraint on misinformation discernment

**DOI:** 10.1038/s41598-026-43330-0

**Published:** 2026-03-23

**Authors:** Yury Shevchenko, Tom Buchanan, Ulf-Dietrich Reips

**Affiliations:** 1 Research Methods, Assessment, and iScience, Department of Psychology, University of Konstanz, Universitätsstraße 10, Fach 31, Konstanz, Germany; 2 School of Social Sciences, University of Westminster, London, UK

Correction to: *Scientific Reports* 10.1038/s41598-026-39555-8, published online 12 February 2026

The original version of this Article contained an error in the name of Yury Shevchenko, which was incorrectly given as Shevchenko Yury.

The original Article has been corrected.

